# Vertical triband flag sign for differential diagnosis of Rathke's cleft cyst

**DOI:** 10.1016/j.wnsx.2023.100260

**Published:** 2023-12-12

**Authors:** Guive Sharifi, Amir Arsalan Amin Darozzarbi, Elham Paraandavaji, Mahmoud Lotfinia, Mohammad Ali Kazemi, Bardia Hajikarimloo, Ali Jafari, Esmaeil Mohammadi, Zahra Davoudi, Nader Akbari Dilmaghani

**Affiliations:** a Department of Neurosurgery, Shahid Beheshti University of Medical Sciences, Tehran, Iran; b Skull Base Research Center, Shahid Beheshti University of Medical Sciences, Tehran, Iran; c Department of Neurosurgery, Medical Center Saarbruecken, Saarland, Germany; d Department of Radiology, Amiralam Hospital, Tehran University of Medical Sciences, Tehran, Iran; e Department of Radiology, Advanced Diagnostic and Interventional Radiology Research Center (ADIR), Medical Imaging Center, Imam Khomeini Hospital Complex, Tehran University of Medical Sciences, Tehran, Iran; f Department of Endocrinology, Shahid Beheshti University of Medical Sciences, Tehran, Iran; g Department of Neurosurgery, University of Oklahoma Health Sciences Center, Oklahoma, USA; h Department of Otolaryngology, Shahid Beheshti University of Medical Sciences, Tehran, Iran

**Keywords:** Rathke's cleft cyst, Magnetic resonance imaging, Diagnosis

## Abstract

**Background:**

The Rathke cleft cyst (RCC) is a type of cystic growth that is benign, circular, and well-defined with an incidence rate of 4 %. This study aims to identify a useful diagnostic imaging sign that can aid in the differentiation of RCC from other cystic lesions.

**Methods:**

We retrospectively analyzed the records of 42 symptomatic RCC patients who were referred to our facility between 2016 and 2023. The data for the study were obtained from our electronic database. All magnetic resonance imaging (MRI) studies were performed using a 1.5-T superconducting magnetic scanner. All patients underwent endonasal transsphenoidal surgical resection. All MRIs were reviewed and evaluated by a neurosurgeon and a neuroradiologist.

**Results:**

There were 8 (19 %) males and 34 (81 %) females with a mean age of 37.2-years. Our study identified a distinct imaging characteristic in 38 of the cases, which we have named the “vertical triband flag sign”, due to the growth of the cyst developing a specific appearance. The flag sign was mostly observed only in the T1-images (71.5 %), while in four cases the sign was spotted only in T2-images, and in four cases it appeared in both T1 and T2. In 4 cases, the flag sign was not observed in which further investigations revealed that these cases were suprasellar or small sellar RCCs. The dot sign, which is a characteristic finding in RCCs was only observed in one of our cases.

**Conclusion:**

Early diagnosis of RCCs may be facilitated by utilizing the vertical triband flag sign.

## 1 Introduction

Rathke's cleft cysts (RCCs) are non-neoplastic cystic lesions of the sellar or suprasellar region that arise from remnants of Rathke's pouch with a 4 % prevalence rate in the general population and 12 %–33 % in autopsy cases.^1^^,^^2^ RCCs can develop in any age group. A previous study demonstrated that the age ranges from 11 to 68 years with an average of 37 years.^3^ As mentioned above, RCCs are sellar or suprasellar lesions that typically develop between the anterior and posterior lobes of the pituitary gland.^4^ RCCs mostly remain asymptomatic but common manifestations include headache, visual loss, visual field deficit, and pituitary endocrine dysfunction due to the compression effect of the lesion on the surrounding tissues.^1^^,^^2^^,^^5^ Meningitis, pituitary abscess, and aneurysm are uncommon clinical manifestations of RCCs.^1^ Depending on their anatomical, clinical, and radiologic characteristics, RCCs may differ in terms of their consistency or texture.^6^

The diagnosis of the RCCs is usually challenging due to the tendency of other cystic lesions such as craniopharyngiomas, arachnoid cysts, epidermoid cysts, and cystic pituitary adenomas to develop at the same anatomical locations as RCCs.^5^ Imaging plays a critical role in the diagnosis and monitoring of RCCs, as differentiating between these various entities is essential for determining the appropriate therapeutic approach.^7^ Magnetic resonance imaging (MRI) is the preferred imaging modality in order to diagnose RCCs.^1^ While several radiological characteristics of RCC have been reported in the literature, their sensitivity is limited. A well-circumscribed, ovoid, and hypoattenuating lesion is characteristic of RCCs in the computed tomography (CT).^5^ The intensity of RCCs may vary case-by-case on MRI, but classically RCCs manifest as T1-hyperintense and T2-hypointense lesions.^5^ Conservative management with follow-up MRI is indicated in asymptomatic or mild symptomic cases while surgical intervention is considered in symptomatic pressure on vital structures.^2^^,^^8^ Transsphenoidal cyst fenestration and drainage is the choice of surgical intervention.^4^^,^^8^ While the gold-standard diagnostic option for RCCs is histopathological finding of cuboidal or columnar epithelium accompanied by cilia, goblet cells, or squamous metaplasia,^5^ it is important to differentiate it from other cystic lesions prior to surgery such as craniopharyngiomas and cystic pituitary adenomas. Imaging techniques provide high-resolution images while failing to completely differentiate between various cystic entities of skull base. Here we tried to identify a new practical radiological sign for RCC that could be easily utilized by radiologists and neurosurgeons using conventional MRI images.

In this study, we aimed to evaluate the demographics, presentations, radiological characteristics, and clinical outcomes of RCC cases regarding the limited literature, especially radiologic features, concurrent with sharing our experience in the management of RCC cases.

## 2 Material and methods

### 2.1 Subjects

In this study, we conducted a retrospective analysis of records from 42 symptomatic RCCs who were referred to our department between 2016 and 2023 and met our inclusion criteria, which required surgical and histological verification of RCC diagnosis. The exclusion criteria were insufficient medical records, previous surgery for RCC, suboptimal MRI images, and absence of contrast agents. The study design was approved by the review board and ethics committee of Shahid Beheshti University of Medical Sciences, and all research adhered to relevant guidelines and regulations. We obtained informed consent from the patients for anonymous use of their data in the study, and for minor subjects under 18 years of age, consent was obtained from their parents or legal guardians.

The data were collected and reviewed by a board-certified and experienced neurosurgeon. The patients were followed-up through telephone calls or in person at the office. All imaging studies were interpreted first by an experienced neurosurgeon and neuroradiologist separately and then the conflicts were evaluated by a second neuroradiologist.

### 2.2 MRI data acquisition

All MRI scans were performed using a 1.5-T superconducting magnetic scanner. The imaging protocol involved obtaining T1-weighted SE and T2-weighted turbo SE images before gadolinium injection, followed by coronal dynamic acquisition (T1-weighted turbo SE) in the coronal plane. Coronal and sagittal T1-weighted SE images were obtained 2-min after the injection. Radiologists and neurosurgeons independently reviewed all imaging studies, including unenhanced and enhanced MRI scans, which were available for all patients. They assessed the cyst's location, size, shape, and signal characteristics. Cyst location and diameter were evaluated on the sagittal MRI. Patients with a midline, homogeneous fluid lesion with little or no enhancement after gadolinium injection were suspected to have RCC.

### 2.3 Diagnosis confirmation

In each case, the diagnosis of RCC was confirmed based on specific histological criteria. These criteria include the presence of dense, amorphous mucin with eosinophilic staining, as well as a cystic wall lining made up of simple cuboidal or pseudostratified columnar epithelium with cilia.

## 3 Results

We admitted 42 symptomatic RCC patients, including 8 (19 %) males and 34 (81 %) females between January 2016 and January 2023, ranging in age from 9 to 74 years old, with a mean age of 37.2 years old (Table 1). As mentioned above, most of the RCCs are asymptomatic but the headache, visual defects, and pituitary dysfunction are the most common manifestations in symptomatic cases and are developed due to compression effect. In our study, headache was the most common presentation and was observed in 24 (57 %) of cases followed by visual deficits. Menstrual irregularities and hyperprolactinemia were the most common endocrinopathies, with a prevalence of 17 % in our study.

**Table 1:** Characteristics of individuals with Rathke cleft cyst.

Case	Sex	Age	Location	T1	T2	Dot sign	Flag sign	Presentation	Outcome
1	F	34	Sellar & Suprasellar	Hypo	Hyper	T2	T1	Amenorrhea – High PRL	Improved
2	F	9	Sellar & Suprasellar	Hypo	Hyper	NA	T1	Headache	Improved
3	F	47	Sellar & Suprasellar	Iso, Hyper	Iso	NA	T1	Amenorrhea - Blurred vision – High PRL	Improved
4	F	42	Suprasellar & Intra stalk	Iso	Iso	NA	T1	Blurred vision – High PRL	Improved
5	F	27	sellar & suprasellar	Hypo	Hyper	NA	T1	Headache	Improved
6	M	47	Sellar & Suprasellar	Hyper	Iso	NA	T1	Asymptomatic	Improved
7	F	30	Sellar & Suprasellar	Hypo	Hyper	NA	T1	Blurred vision	Improved
8	F	60	Sellar & Suprasellar	Iso	Hyper	NA	Irregular^a^	DI	Improved
9	M	22	Sellar	Hyper	Hyper	NA	NA	Headache - Delayed puberty – High Tes – Low cortisol	Improved
10	M	37	Sellar	Hypo	Iso	NA	T1	incidental	Improved
11	F	35	Sellar	Hyper	Hypo	NA	T1, T2	Bitemporal hemianopia	Improved
12	F	56	Sellar	Hyper	Hypo	NA	T2	Pan-hypopituitarism	Improved
13	F	21	Sellar	Iso	Hyper	NA	T1	Headache -Blurred vision	Improved
14	F	36	Sellar	Hyper	Iso	NA	T1	Bitemporal hemianopia	Improved
15	F	42	Sellar & Suprasellar	Hyper	Hyper	NA	T1	Headache – High PRL	Improved - Slightly elevated PRL
16	F	47	Sellar	Iso	Iso	NA	T1	Headache – High PRL	Improved - Slightly elevated PRL
17	F	42	Sellar	Hypo	Hyper	NA	T1	Headache	Improved
18	F	34	Sellar & Suprasellar	Hyper	Hyper	NA	NA	Headache – DI – High PRL	Improved
19	F	42	Sellar	Hypo	Hyper	NA	T1	Amenorrhea – High PRL	Improved
20	F	33	Sellar	Iso, Hypo	Hyper	NA	T1	Amenorrhea -DI – High PRL	Improved
21	M	28	Sellar	Hyper	Hypo	NA	T1, T2	Headache -Blurred vision - Impotency – High PRL	Improved
22	F	35	Sellar	Hyper	Hypo	NA	T2	Blurred vision	Improved
23	M	71	Sellar & Suprasellar	Hyper	Hyper	NA	T1	Headache - Blurred vision	Improved
24	F	46	Suprasellar	Iso, Hypo	Iso, Hyper	NA	NA	Blurred vision	Improved
25	M	74	Sellar & Suprasellar	Iso, Hypo	Iso, Hyper	NA	T1	Headache - Blurred vision	Improved – CSF leak
26	F	44	Sellar	Iso, Hyper	Iso, Hypo	NA	T1	Headache	Improved – CSF leak
27	F	39	Sellar	Hyper	Hypo	NA	T2	Headache	Improved
28	F	36	Sellar & Suprasellar	Hyper	Hyper	NA	T1	Headache	Improved
29	F	30	Sellar & Suprasellar	Hyper	Iso	NA	T1	Headache	Improved
30	F	36	Sellar & Suprasellar	Iso, Hyper	Hyper	NA	T1	Headache	Improved
31	F	41	Sellar & Suprasellar	Hyper	Hyper	NA	T1	Headache - Blurred vision	Improved
32	F	31	Sellar & Suprasellar	Hyper	Hypo	NA	T1	Headache - Blurred vision	Improved
33	F	36	Sellar & Suprasellar	Hyper	Iso, Hyper	NA	T1	Headache	Improved
34	M	28	Sellar & Suprasellar	Hyper	Iso, Hyper	NA	NA	Headache -Blurred vision - Hyperthyroidism	Improved
35	M	28	Sellar & Suprasellar	Hyper	Hypo	NA	T1, T2	Impotency -DI	Improved
36	F	37	Sellar & Suprasellar	Hyper	Iso	NA	T1	Headache -Diplopia	Improved
37	F	29	Sellar & Suprasellar	Hyper	Hyper	NA	T1	Headache -Diplopia	Improved
38	F	39	Sellar	Hyper	Hypo	NA	T1, T2	Headache - Blurred vision	Improved
39	F	25	Sellar & Suprasellar	Hyper	Hypo	NA	T2	Blurred vision	Improved
40	F	25	Sellar & Suprasellar	Iso	Iso	NA	T1	Menstural disorder – Galactorrhea – High PRL -Low cortisol	Improved
41	F	38	Sellar & Suprasellar	Hypo	Hyper	NA	Irregular^a^	Headache	Improved
42	F	25	Sellar & Suprasellar	Hyper	Iso, Hyper	NA	T1	Amenorrhea - DI	Improved

DI = Diabetes insipidus PRL= Prolactinoma NL= Normal Tes = Testosterone NA=Not available T1 = T1-weighted images T2 = T2-weighted images.

a In these cases, the flag sign was observed but despite other cases, this sign had irregular borders.

RCCs usually develop in sellar and suprasellar regions. In our study, in 15 (36 %) of cases, the lesion was located in the sellar region; in 2 (5 %) it was located in the suprasellar region; and 25 (59 %) cases, the lesion was placed in the sellar region with suprasellar extension.

While the typical appearance of symptomatic RCC is commonly reported as iso- tohypointense on T1-weighted images and hyperintense on T2-weighted images, and isointense on CT scans, we observed a variety of intensities in our cases (Table 1). In 5 (12 %) of cases the lesion was isointense in T1, in 3 (7 %) was iso-hypointense, in 3 (7 %) was iso-hyperintense, and in 8 (19 %) and 23 (55 %) was hypointense and hyperintense, respectively. In the setting of T2 images, in 9 (21 %) of cases the lesion was isointense, in 1 (2 %) was iso-hypointense, in 5 (12 %) was iso-hyperintense, and in 9 (21 %) and 18 (44 %) was hypointense and hyperintense, respectively (Table 1).

**Fig. 1:**
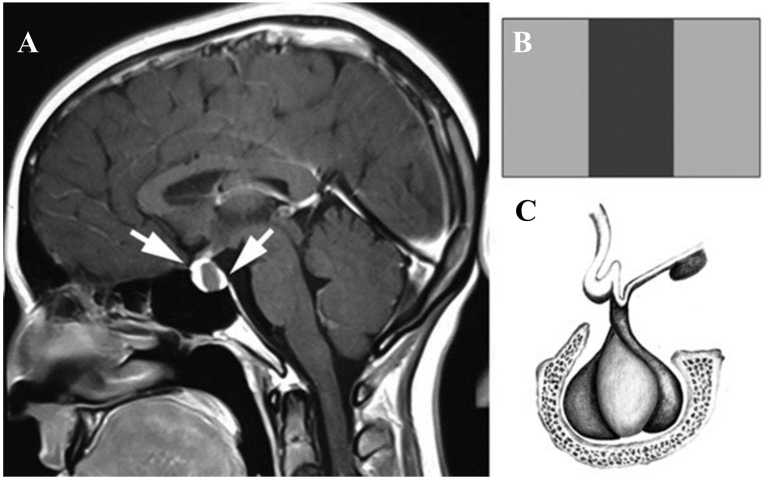
The Vertical Triband Flag sign was observed in 38 individuals with RCC in our study. The sign was mostly observed only in the T1 images (71.5 %), while in four cases the sign was spotted only in T2 images, and in four cases it appeared in both T1 and T2 images. The flag sign appeared as a hyperintense sign in T1 and a hypointense sign in T2 images due to the high density of the fluid inside the cyst. In 4 (9.5 %) cases the flag sign was not observed.

**Fig. 2:**
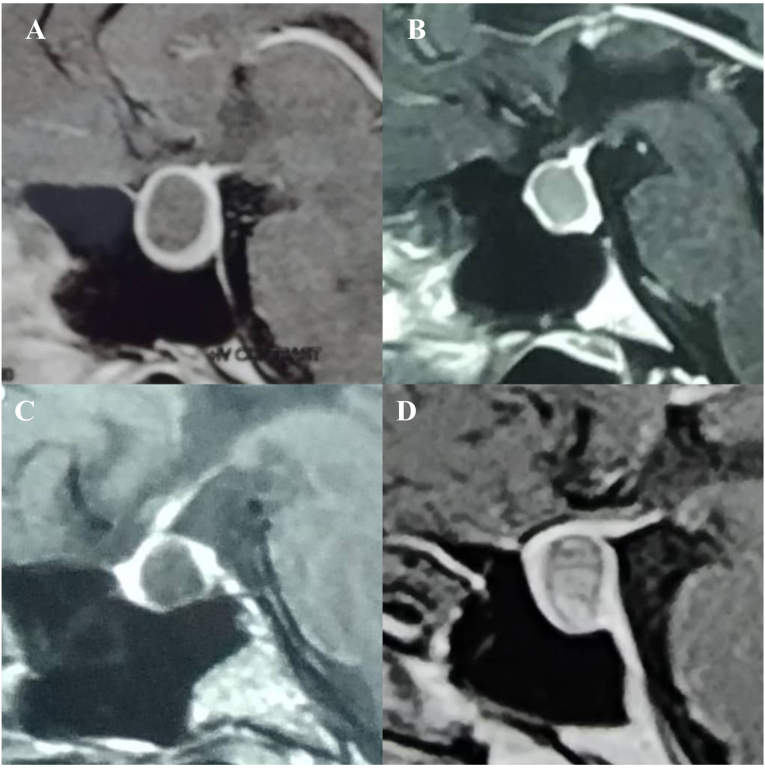
This figure demonstrates the presence of the Vertical Triband Flag sign in T1 images. In our study, in most of the cases, the sign was observed only in T1 images with a prevalence of 71.5 %.

**Fig. 3:**
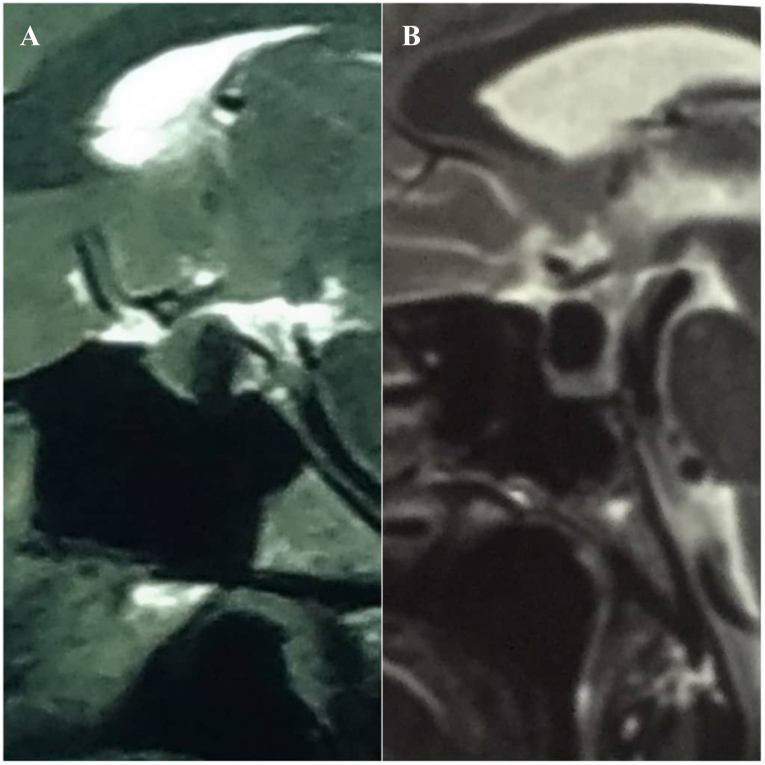
This figure demonstrates the presence of the Vertical Triband Flag sign in T2 images. In our study, in only four individuals with RCC, the sign was observed merely in T2 images.

**Fig. 4:**
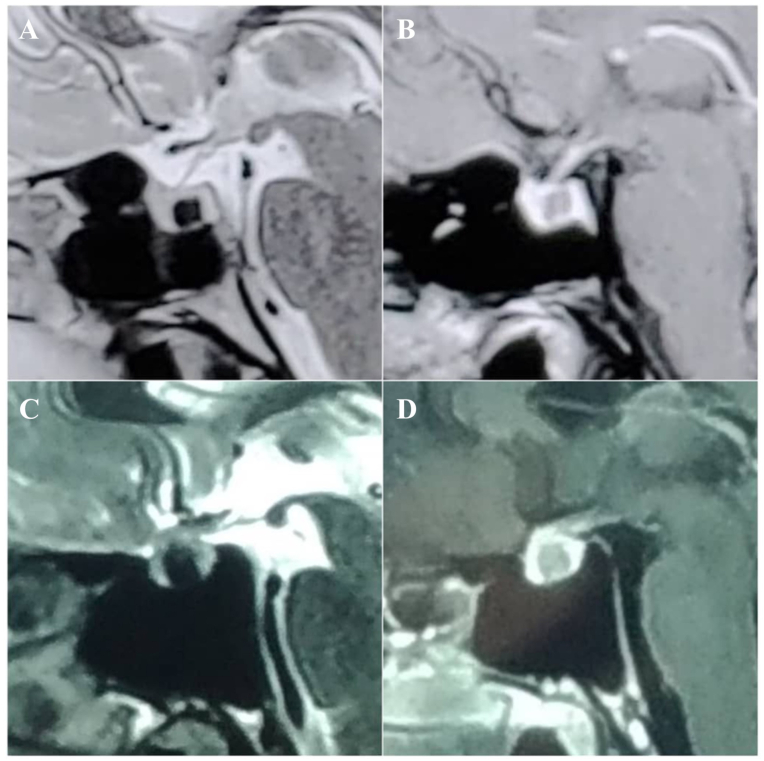
This figure demonstrates the cases that the flag sign was observed in both T1 and T2 images and this condition was observed in four individuals in our study.

RCC grows between the adenohypophysis and neurohypophysis, pushing the former towards the front and the latter towards the back. This process results in the gland's enhancement on the floor, anterior and posterior walls of the sella turcica, producing a schematic appearance of three vertical bands that resemble a flag. We named this the "vertical triband flag sign." In our sample, in 38 (90.5 %) cases this pattern was observed (Fig. 1). In our study, the flag sign was mostly observed only in the T1 images (71.5 %) (Fig. 2), while in four cases the sign was spotted only in T2 images (Fig. 3), and in four cases it appeared in both T1 and T2 images (Fig. 4). The flag sign appeared as a hyperintense sign in T1 and a hypointense sign in T2 images due to the high density of the fluid inside the cyst.

**Fig. 5:**
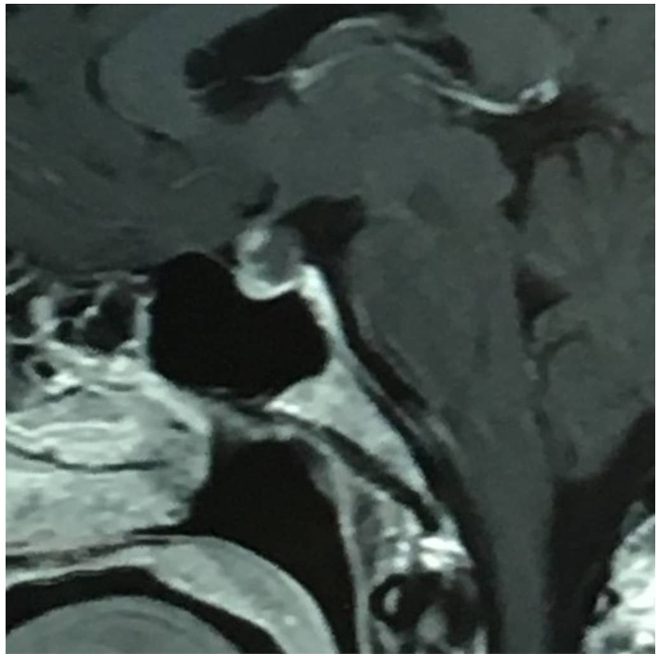
This figure demonstrates the irregular border of the flag sign. Despite observing regular borders in 36 cases, the borders were irregular in two cases, which is a main characteristic of cystic pituitary adenomas. Further investigations revealed that in these two cases, according to the prolonged and chronic course of the disease, the inflammation had extended into the adenohypophysis, similar to cystic adenoma cases, and resulted in the formation of irregular borders.

Regarding the variability in volume of the lesion and compression effect, the borders are not necessarily formed in a straight manner. However, the borders should possess a regular appearance. Despite observing regular borders in 36 cases, the borders were irregular in two cases, which is a main characteristic of cystic pituitary adenomas. Further investigations revealed that in these two cases, according to the prolonged and chronic course of the disease, the inflammation had extended into the adenohypophysis, similar to cystic adenoma cases, and resulted in the formation of irregular borders (Fig. 5).

In 4 (9.5 %) cases the flag sign was not observed. Further investigation suggested that the flag sign does not appear in suprasellar or small sellar RCCs; therefore, in these conditions, other characteristics should be evaluated.

One of the characteristic findings of the RCC lesions is a hypointense dot sign within the cyst. In our study, the dot sign was only observed in one of our cases, which demonstrated a significantly lower prevalence compared with the flag sign.

All patients underwent endonasal transsphenoidal surgical resection of the lesion. No major complication was observed intraoperatively. Headache and visual impairment resolved in all of the patients postoperatively. Two individuals developed cerebrospinal fluid leaks and underwent second endonasal transsphenoidal surgery; consequently, the leak was repaired in one case, and in the other, the leak did not resolve, and a ventriculoperitoneal shunt was placed. Endocrinopathies were observed in 13 (31 %) of individuals preoperatively and resolved in 11 patients, and only two patients had persistent asymptomatic hyperprolactinemia subsequent to the surgery.

**Illustrative case:** A 42-year-old woman with a diagnosis of prolactinoma presented with dysmenorrhea and was treated with cabergoline therapy by an endocrinologist. A sagittal magnetic resonance image with gadolinium injection revealed a cyst growing between the anterior and posterior pituitary gland, which caused enhancement of the gland, resulting in the characteristic appearance of the vertical triband flag sign on imaging (Fig. 1). The arrows on the image mark the enhanced posterior and anterior pituitary gland. Despite being refractory to treatment, the patient underwent surgery and was found to have RCC upon pathological examination.

## 4 Discussion

The preoperative assessment of RCCs and differentiation from other cystic sellar lesions can be effectively achieved using MRI, which allows for the examination of various features such as shape and signal intensity. However, the diverse appearance of RCCs on MRI can make their neuroimaging diagnosis challenging.^9^ The T1 usually reveals low intensity when the protein concentration is low, while high intensity is observed when the protein concentration is high.^10^ The typical MRI signal patterns for RCC can be any of the following combinations: hypointensity on T1 and hyperintensity on T2; isointensity on T1 and hyperintensity on T2; or hyperintensity on both T1 and T2.^11^ The majority of cases show little or no enhancement of the cyst wall or contents on MRI images after Gadolinium administration. However, in some cases, a thin enhancing rim may be observed, which is believed to be a consequence of inflammation, squamous metaplasia of the cyst wall, or a rim of displaced hypophysis.^3^ According to Chotai et al, the low signal intensity of the cystic content on T2 can be a significant predictor of recurrence, even when other imaging features are taken into account.^12^ According to Wajima et al, there are two types of RCCs based on their signal intensity on T2. Lesions with hypointensity on T1 and hyperintensity on T2 are classified as Type 1, while all other types of appearance on imaging fall under Type 2.^13^ The authors proposed that lesions classified as type 1, which exhibit hypointensity on T1 and hyperintensity on T2, tend to have a more aggressive course and grow more rapidly than type 2 lesions, which exhibit other types of appearance on imaging and are generally more indolent. It's possible for an intracystic nodule to be found in more than 40 %–75 % of RCCs that exhibit a hyperintense lesion on T1 and a hypointense lesion on T2.^14^ In our study, despite previous studies, the predominant intensity patterns were hyperintense in both T1 and T2 images. Regarding location and size, the majority of cases were primarily located in the sella turcica (36 %), with only 2 of the 42 cases being primarily suprasellar. Of the 42 intrasellar cysts, 25 (59 %) had a secondary suprasellar extension. In the preoperative evaluation of patients with cystic sellar lesions, it is crucial to differentiate RCC from other conditions, particularly cystic pituitary adenomas and craniopharyngiomas. The presence of a fluid–fluid level, hypointense rim on T2, septation, and off-midline location are more commonly seen in pituitary adenomas, while the presence of an intracystic nodule is more commonly observed in RCC.^15^ Craniopharyngiomas are another significant differential diagnosis for RCCs. They typically have an irregular shape, are large, and are located in the suprasellar region. On CT scans, they often appear as homogeneous, low-density cysts without enhancement, although isodensity, high-density, and calcification have also been reported.^16^ Another distinguishing feature of RCCs is central location without stalk deviation and T2 hypointensity.^5^

We observed the growth of cyst material between the adenohypophysis and neurohypophysis, resulting in the thrust of the adenohypophysis towards the anterior and the neurohypophysis towards the posterior. As a result, in sagittal sections, gland enhancement can be observed on the anterior, floor, and posterior wall of the sella turcica, which resembles a vertical triband flag. In our department, we have not seen this imaging pattern in sellar lesions except in cases of RCC, among the 1351 patients who underwent surgery since 2016 at the time of drafting this manuscript. We also could not find any reports of a similar appearance on MRI for either RCC or other brain lesions. Due to the ability to mimic other intrasellar or suprasellar masses, such as cystic pituitary adenoma, abscess, craniopharyngioma, and arachnoid cyst, the diagnosis of RCCs can be challenging. Especially in some cases, RCC cannot be distinguished from cystic adenoma. In this series of RCC patients, 38 (90.5 %) exhibited this highly specific sign on MRI. Although we are aware of its intermediate sensitivity, we wish to introduce this sign as a valuable diagnostic feature of RCC. The flag sign most commonly appeared only in the T1 images with a prevalence of 71.5 %(Fig. 2), while in four cases the sign was seen only in T2 images (Fig. 3), and in four cases it appeared in both T1 and T2 images (Fig. 4). The flag sign appeared in most of the individuals with regular borders; however, irregular borders were observed in two cases, which is a main characteristic of cystic pituitary adenomas. Subsequent investigations revealed due to the prolonged and chronic course of the disease, the inflammation had extended into the adenohypophysis, similar to cystic adenoma cases, and resulted in the formation of irregular borders in these two cases.

The flag sign can be utilized routinely as a diagnostic sign in the diagnosis of RCCs and help physicians differentiate RCCs from other lesions with similar characteristics, especially consudering the fact that this sign is only observed in RCC individuals with a high prevalence. As mentioned above, cystic pituitary adenoma is one of the most important differential diagnoses of RCCs. The regular border of the flag sign is an important differentiating feature and can be used to distinguish RCCs from cystic adenomas. In addition, the dot sign, which has been described as a characteristic feature of RCCs in the literature, was only observed in one of our cases; however, the flag sign was observed in 90 % of cases. Therefore, we suggest the vertical triband flag sign as a unique radiological and differentiating feature of the RCCs that can be applied routinely in the establishment of the diagnosis of RCCs in order to increase the accuracy of the diagnosis.

### 4.1 Limitations

Our study has several limitations. The retrospective nature of our study is the first limitation of our study due to the fact that retrospective studies interpret the information that has already been gathered. The second limitation is that despite the valuable data that we presented, the size of our study is limited and this limitation can influence the power of our study. Furthermore, the study was conducted at a single center, and due to the referral nature of our institute, findings may have been influenced by the selection of more complicated patients. Therefore, it may not be possible to generalize our findings to every patient population. We also believe that a larger study with robust statistical analysis may provide more definitive results and could validate the sign in the future.

## 5 Conclusion

The presence of the vertical triband flag sign on MRI can be used as a radiological feature for the early diagnosis of RCC patients. Therefore, neurosurgeons should be aware of cystic lesions of the skull base and have various differential diagnoses in mind. However, further validation is needed to determine the exact specificity and sensitivity of this sign, which should be investigated in larger and multi-center studies.

## Funding

No funding or financial support was received for this study.

## CRediT authorship contribution statement

**Guive Sharifi:** Writing - review & editing, Conceptualization. **Amir Arsalan Amin Darozzarbi:** Writing - review & editing, Conceptualization. **Elham Paraandavaji:** Writing - review & editing, Writing - original draft. **Mahmoud Lotfinia:** Writing - review & editing. **Mohammad Ali Kazemi:** Investigation, Methodology, Visualization. **Bardia Hajikarimloo:** Writing - review & editing. **Ali Jafari:** Resources. **Esmaeil Mohammadi:** Writing - review & editing, Methodology. **Zahra Davoudi:** Writing - original draft. **Nader Akbari Dilmaghani:** Supervision.

## Declaration of competing interest

The authors declare that they have no known competing financial interests or personal relationships that could have appeared to influence the work reported in this paper.
